# Associations Between *IGF1, IGFBP2* and *TGFß3* Genes Polymorphisms and Growth Performance of Broiler Chicken Lines

**DOI:** 10.3390/ani10050800

**Published:** 2020-05-05

**Authors:** Bozena Hosnedlova, Katerina Vernerova, Rene Kizek, Riccardo Bozzi, Jaromir Kadlec, Vladislav Curn, Frantisek Kouba, Carlos Fernandez, Vlastislav Machander, Hana Horna

**Affiliations:** 1 Veterinary Research Institute, Hudcova 296/70, 621 00 Brno, Czech Republic; kizek@sci.muni.cz; 2 Biotechnological Centre, Faculty of Agriculture, University of South Bohemia in České Budějovice, Studentská 1668, 370 05 České Budějovice, Czech Republic; kate.vernerova@gmail.com (K.V.); vcurn@seznam.cz (V.C.); 3 Department of Human Pharmacology and Toxicology, Faculty of Pharmacy, University of Veterinary and Pharmaceutical Sciences Brno, Palackého 1946/1, 612 42 Brno, Czech Republic; 4 Department of Biomedical and Environmental Analyses, Faculty of Pharmacy with Division of Laboratory Medicine, Wroclaw Medical University, Borowska 211, 50-556 Wroclaw, Poland; 5 Food, Environment and Forestry, Animal Science Section, Department of Agriculture, University of Florence, Via delle Cascine, 5, 50144 Firenze, Italy; riccardo.bozzi@unifi.it; 6 Department of Agricultural Products’ Quality, Faculty of Agriculture, University of South Bohemia in České Budějovice, Studentská 1668, 370 05 České Budějovice, Czech Republic; kadlec@zf.jcu.cz; 7 State Veterinary Administration, Regional Veterinary Administration of the South Bohemian Region, Severní 9, 370 10 České Budějovice, Czech Republic; f.kouba.kvsc@svscr.cz; 8 School of Pharmacy and Life Sciences, Robert Gordon University, Garthdee Road, Aberdeen AB10 7QB, UK; c.fernandez@rgu.ac.uk; 9 International Testing of Poultry, Ústrašice 63, 390 02 Tábor, Czech Republic; vlastislav.machander@mtd-ustrasice.cz (V.M.); hana.horna@mtd-ustrasice.cz (H.H.)

**Keywords:** chicken, SNP, *IGF1*, IGFBP2, TGFß3, Hubbard F15, Cobb E, growth, meat

## Abstract

**Simple Summary:**

The main goal of breeding programs for broiler chickens is to increase growth rate and breast and thigh muscles weight. The candidate gene approach is a powerful technique for genetically improving performance traits in chickens. We studied the associations of the single nucleotide polymorphisms of three genes involved in protein synthesis, glucose metabolism and cell proliferation (*IGF1, IGFBP2, TGFβ3*) with performance traits in the Hubbard F15 and Cobb E chicken lines. Based on our results, it can be concluded that the *TGFβ3* gene could be used as a candidate gene marker for chicken growth traits.

**Abstract:**

Marker-assisted selection based on fast and accurate molecular analysis of individual genes is considered an acceptable tool in the speed-up of the genetic improvement of production performance in chickens. The objective of this study was to detect the single nucleotide polymorphisms (SNPs) in the *IGF1, IGFBP2* and *TGFß3* genes, and to investigate their associations with growth performance (body weight (BW) and average daily gain (ADG) at 14, 21, 28, 35 and 42 days of age) and carcass traits in broilers. Performance (carcass) data (weight before slaughter; weights of the trunk, giblets, abdominal fat, breast muscle and thigh muscle; slaughter value and slaughter percentage), as well as blood samples for DNA extraction and SNP analysis, were obtained from 97 chickens belonging to two different lines (Hubbard F15 and Cobb E) equally divided between the two sexes. The genotypes were detected using polymerase chain reaction- restriction fragment length polymorphism (PCR-RFLP) methods with specific primers and restrictase for each gene. The statistical analysis discovered significant associations (*p* < 0.05) between the *TGFβ3* SNP and the following parameters: BW at 21, 28 and 35 days, trunk weight and slaughter value. Association analysis of BWs (at 21, 28 and 35 days) and SNPs was always significant for codominant, dominant and overdominant genetic models, showing a possible path for genomic selection in these chicken lines. Slaughter value was significant for codominant, recessive and overdominant patterns, whereas other carcass traits were not influenced by SNPs. Based on the results of this study, we suggested that the *TGFβ3* gene could be used as a candidate gene marker for chicken growth traits in the Hubbard F15 and Cobb E population selection programs, whereas for carcass traits further investigation is needed.

## 1. Introduction

With the growth of the human population [1], the total amount of meat consumed increases at a global level worldwide [2]. Meat consumption rose worldwide from 23.08 kg per person per year in 1961 to 43.22 kg per person per year in 2013 [2]. Chicken meat is one of the most consumed types of meat worldwide [3].

Growth performance and carcass traits are the most important economic traits in broiler chicken production, and are controlled by a number of genes [4]. Growth is a complex process that involves the regulated coordination of a wide range of neuroendocrine pathways [5]. Therefore, it is very difficult to achieve rapid genetic improvement in these traits using only traditional selection methods. The growing knowledge of the structure and function of the chicken genome can be beneficial, and can lead to the recognition of causal genes and the development of new selectable molecular markers.

Although the *Gallus gallus* (chicken) genome was first sequenced as early as 2004 [6], it still required further improvements [7,8,9]. The newest version of the chicken genome assembly (Gallus_gallus-5.0; GCA_000002315.3), built from combined long single molecule sequencing technology, finished bacterial artificial chromosomes (BACs) and improved physical maps, was presented in 2017 [10]. Since the methodological approach has improved, the originally reported size of the chicken genome has increased from 1.05 [6] to 1.23 Gb, which has contributed to the increased number of genes observed [10]. Initial assemblies have been found insufficient for the more complete discovery of allelic contributions to complex traits [10], leading to ongoing efforts to improve the quality of the chicken reference genome [8,11].

However, the genetic improvement of polygenic traits, including growth performance and meat production, can be accomplished by marker-assisted selection that is more accurate in estimating the animal’s genetic value [12]. The molecular markers linked to quantitative trait loci (QTLs) are not affected by environmental conditions. Therefore, they could increase the speed and effectiveness of animal breeding progress. As soon as the relationship between a DNA polymorphism and an important trait is revealed, the DNA marker may be used [13]. The candidate gene approach has become a powerful technique for the genetic improvement in chicken breeding programs, and can result in increased efficiency in detecting the required production performance traits [4]. 

The main objectives of the strategy in commercial broiler breeding programs include increasing growth rate and breast muscle weight, reducing abdominal fat content, improved feed efficiency and increased fitness. The relationships between these individual production traits are very complex and some of them are very difficult to measure. Therefore, the use of molecular marker-assisted selection (MMAS) is necessary. In case that the favorable allele is rare, a larger positive impact can be expected [14]. 

**Figure 1:**
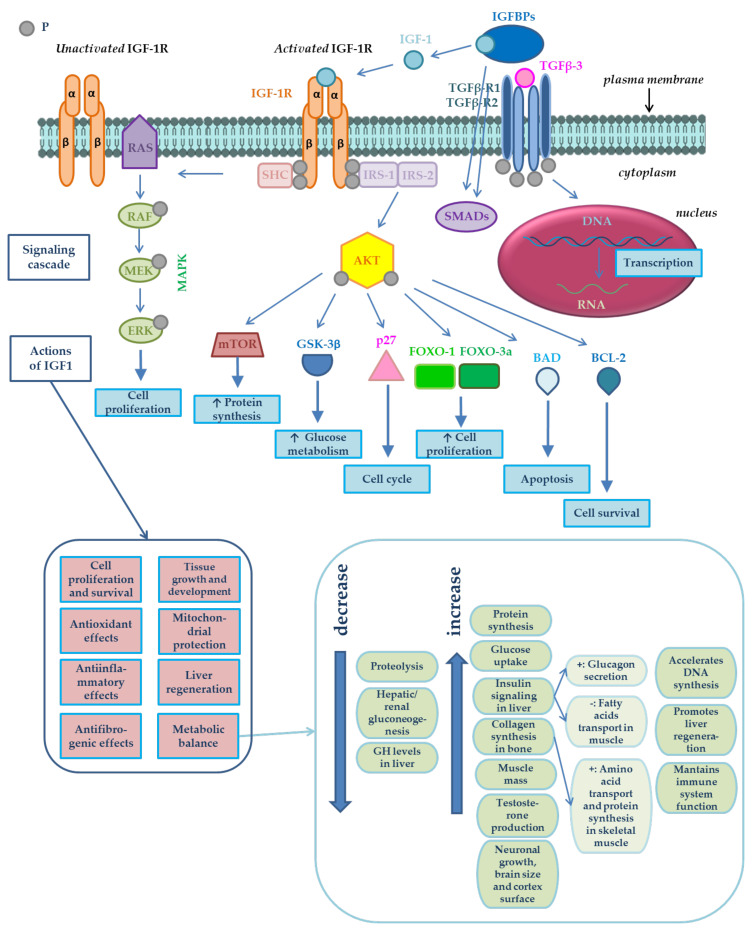
Signaling cascade of insulin-like growth factor 1 (IGF-1) and its potential impacts in metabolism, its interactions with transforming growth factor β3 (TGF-β3) and the biological functions of the *IGF1*, *IGFBP2* and *TGFß3* genes. IGF-1 bioavailability is modulated by IGF binding proteins (IGFBPs) [15]. IGF-1 action is mediated by its binding to its receptor [16], the type 1 insulin-like growth factor receptor (IGF-1R). IGF-1R is a heterotetramer composed of two extracellular α subunits and two transmembrane β subunits, as shown in Figure 1. α subunits are cysteine-rich regions, whereas β subunits possess a tyrosine kinase domain, which constitutes the signal transduction mechanism [16]. Tyrosine phosphorylation activates a signaling cascade [17]. IGF-1 has autocrine, paracrine [18,19] and endocrine effects [18]. IGF-1 binds to its receptor (IGF-1R) in the cell membrane, resulting in autophosphorylation and the recruitment of the adaptor proteins–insulin receptor substrate IRS-1, IRS-2, and the proto-oncogene tyrosine-protein kinase (SRC) homology and collagen protein (SHC). The serine/threonine kinase (AKT) is activated by the 3-phosphoinositide-dependent protein kinase-1 (PDK1) and by the mammalian target of rapamycin (mTOR)-containing complex mTOR-C2, leading to the phosphorylation at threonine 308 and serine 473, respectively. Activated AKT regulates downstream signaling molecules such as tuberous sclerosis protein 1/2 (TSC-1/2), which inhibit mTOR-C1 complex and regulate the ribosomal protein S6 kinase 1/2 (S6K-1/2) and eukaryotic translation initiation factor 4E (eIF4E)-binding protein 1 (4EB-P1) phosphorylation, FOXO transcription factors, glycogen synthase kinase-3β (GSK-3β), p27, BCL-2 antagonist of cell death (BAD), and BCL-2. These substances are involved in some cellular processes such as protein synthesis, glucose metabolism and cell survival. SHC activation induces the activation of the RAS/mitogen-activated protein (MAP) kinase pathway, resulting in enhanced cell proliferation [15]. Activation of IRS induces the activation of intracellular RAF/MEK/ERK/RAS and PI3K signaling pathway. The first mentioned pathway mediates mitosis, and the second one mediates metabolism and cell growth effect through AKT [20]. After the ligand (IGF-1) binds to its receptor (IGF-1R), PI3K is activated, cell proliferation is promoted by activating the mitogen-activated protein kinase (MAPK) cascade, and apoptosis is blocked by inducing the phosphorylation and the inhibition of proapoptotic proteins such as BAD [21]. The protein IGFBP-2 encoded by the gene of the same name is able to control the biological actions of IGFs [22] and TGFß [23] in vivo via the endocrine, autocrine or paracrine pathways. The protein TGFß-3 encoded by the *TGFβ3* gene controls the growth, proliferation and differentiation of cells, cell motility and apoptosis. TGFß-3 plays an essential role in the development of skeletal muscles. It also can suppress the formation of tumors [24]. Adapted from [15,25,26,27] based on other works: [21,28,29,30,31,32,33,34,35,36,37,38,39,40,41,42,43,44,45,46,47,48,49,50,51,52,53].

The purpose of the present study was to identify polymorphisms and evaluate the association between polymorphisms in three studied genes—*IGF1* (insulin-like growth factor 1), *IGFBP2* (insulin-like growth factor binding protein 2) and *TGFß3* (transforming growth factor β)—with growth performance and meat production in chickens from two broiler lines: Hubbard F15 and Cobb E. The biological functions and interdependence of these genes are shown in Figure 1.

The insulin-like growth factor 1 gene (*IGF1*) has been identified as a biological candidate gene for growth, body composition, metabolic and skeletal characteristics, and is also a positional candidate gene for growth and fat deposition in chicken [54]. This gene is involved in growth of various tissues such as muscle and bones [55]. The chicken *IGF1* gene was mapped to 165.95 cM on chromosome 1 (GGA1). In a broiler-layer F_2_ population used to map body weight (BW) QTL by a genome scan, a QTL affecting BW at 6 weeks of age has been detected at 160 cM (confidence interval (CI) 114 to 180 cM) on chicken GGA1 [56]. In the same F_2_ cross, a QTL at 150 cM (CI from 100 to 182 cM) on GGA1 affecting abdominal fat weight (AFW) has been ascertained [57]. 

The bioavailability of the insulin-like growth factors (IGFs) is regulated by a family of structurally conserved insulin-like growth factor binding proteins (IGF-binding proteins; IGFBPs) [58,59,60]. IGFBPs selectively bind to IGF-1 and IGF-2 proteins but do not bind to insulin [61]. More than 99% of IGF molecules circulate in blood serum as complexes with these specific and high affinity-binding proteins. Although IGF-binding protein 3 (IGFBP-3) is a main component and binds over 75% of the circulating IGF [62], IGFBP-2 is sensitive to dietary protein level, and may play a substantial role in the modulation of the growth-promoting effect of circulating IGF-1 by creating the IGF-1‒IGFBP-2 complex in chickens [63]. IGFBP-2 is the predominant IGFBP in serum for different species [64]. IGFs, IGFBPs and IGFBP proteases are the major regulators of somatic growth and cell proliferation [65]. IGFBP-2 controls the biological actions of IGFs [22] and TGFs [23] in vivo via endocrine [22,23], autocrine [23] or paracrine mechanisms [22], and affects the growth and development of animals [66]. IGFBP-2 might indirectly affect adipocyte differentiation by controlling IGF [67] and TGF-β biological actions in fat tissue [68]. 

The transforming growth factor β (TGF-β) belongs to a large family of multifunctional growth factors [69], with important regulatory roles in embryonic and adult development [70], such as morphogenesis, development and differentiation [69]. Polypeptide growth factors of TGF-β family regulate a number of cellular processes such as cell proliferation, differentiation, migration, adhesion and apoptosis [70]. TGF-β plays a key role in maintaining both bone and articular cartilage homeostasis [71]. 

## 2. Materials and Methods 

### 2.1. Experimental Population—Animals 

The chicken hatching eggs were produced and the experiment was performed in the testing station of broilers (fattening test No. 1148) at the state-owned enterprise International Testing of Poultry, Ustrasice (Czech Republic). After hatching, 50 chickens from each of the two broiler lines Hubbard F15 and Cobb E were stalled in windowless air-conditioned hall with deep bedding and controlled light mode (Table 1). Stocking density was 6.1 chicks/m^2^. The hall was disinfected with Virkon before the chickens were stored. The chickens were watered by automatic dropper drinking basins and fed with three feed mixtures, differently for particular period of fattening, from tube feeders *ad libitum*. Hypermangan solution was applied to the water in the first days of age. The composition (contents of main nutrients) in individual complete feed mixtures BR1, BR2 and BR3 for fattening broiler chickens up to the 10th, 35th and 42nd day of age, respectively, are shown in Table 2. 

### 2.2. Phenotypic Data

Body weight (BW) was measured at 14, 21, 28, 35 days and before slaughter at 42 days of age. The mortality during experiment was 3% (sudden death syndrome). Chickens were slaughtered at 42 days of age and the slaughter analysis was performed. The carcass traits, such as weight of trunk, giblets, abdominal fat, breast muscle (with and without skin) and thigh muscle (with and without skin), as well as slaughter value and slaughter percentage were investigated. The slaughter value was calculated as the ratio between the weight of the carcass (trunk weight) and the weight at 42 days of age before slaughter, and the slaughter percentage was calculated as the ratio between the sum of weight of the trunk and giblets and the weight at 42 days.

### 2.3. SNP Genotyping

#### 2.3.1. DNA Extraction 

Genomic DNA for genotyping assays was extracted from whole blood samples, which were collected from 97 chickens at 42 days of age before slaughtering. Blood was taken from *vena ulnaris* into 1.5 mL EDTA-treated microtubes. For extraction of genomic DNA, chelex 100 was used, and the concentration and purity of genomic DNA were verified by spectrophotometer Shimadzu BioSpec-nano (Shimadzu Corporation, Kyoto, Japan). 

#### 2.3.2. Optimalization of PCR-RFLP Assay

Polymerase chain reaction (PCR) was performed for all assays in total volume 25 µL mixture containing 1 µL genomic DNA, 10 pmol of each primer and 12.5 µL PPP Master Mix (Top-Bio, s.r.o., Vestec, Czech Republic). Sequences of sets of primer pairs for all three gene polymorphisms used in PCR assays are shown in Table 3.The primers for the *IGF1* genotyping were designed according to Moody et al. [72]. The thermal profile included pre-denaturation at 94 °C for 2 min followed by 30 cycles 94 °C for 30 s, 67 °C for 30 s and 72 °C for 50 s, with a final extension of 72 °C for 7 min. Thermocycler BIOER Life ECO (Hangzhou Bioer Technology Co,. Ltd., Bin An Rd, Hi-tech (Binjiang) District, Hangzhou, China) was used for DNA amplification. SNP of *IGF1* gene was detected after digesting PCR product with *Hinf*I restriction endonuclease (Fermentas, Vilnius, Lithuania) at 37 °C overnight. For detection of *IGFBP2* genotypes PCR amplification was done using primer set by Li et al. [73]. Amplification was performed under following conditions: pre-denaturation at 94 °C for 2 min followed by 30 cycles 94 °C for 30 s, 54 °C for 30 s and 72 °C for 30 s, with a final extension of 72 °C for 7 min. PCR products were digested (restriction fragment length polymorphism–RFLP) with *Eco72*I restriction endonuclease (New England Biolabs, Ipswich, MA, USA) at 37 °C overnight. The PCR primers designed by Li et al. [69] were applied for *TGFβ3.* The PCR reaction conditions were the same as for *IGFBP2*, except the annealing temperature which was 58 °C. Gene fragments were subjected to digestion by *Bsl*I restriction enzyme (New England Biolabs, Ipswich, MA, USA) at 37 °C overnight. 

#### 2.3.3. Electrophoresis

The PCR products were visualized by 2% and restriction patterns by 3% agarose gel electrophoresis and ethidium bromide staining. The ENDURO™ 250 V power supply (Labnet International, Inc., New York, USA) and the HU13 midi horizontal gel electrophoresis unit (Scie-Plas Ltd., Cambourne, Cambridge, UK) were used for DNA electrophoresis. Syngene™ Ingenius 3 Manual Gel Documentation System (Syngene) was used for photo-documentation.

### 2.4. Statistical Analysis

Associations of three different polymorphisms of *IGF1*, *IGFBP2* and *TGFβ3* genes with growth characteristics and carcass data in 97 chicken belonging to two different lines (Hubbard F15 and Cobb E), equally divided between two sexes, were studied. Genotypes were tested for Hardy-Weinberg equilibrium (HWE) using a chi-square (χ^2^) test in R/SNPassoc Package (R Development Core Team). Whole-Genome association analyses were performed assuming five different genetic models (inheritance patterns) using R/SNPassoc Package (R Development Core Team): codominant, dominant, recessive, overdominant and log-additive effect. The level of significance was tested at the nominal 5% significance level after correcting for the number of tests performed (Bonferroni correction). Phenotypes were represented by the carcass data collected on poultry, whereas line and sex were included in the model as fixed effects. Hardy-Weinberg equilibrium (HWE) was calculated and tested using χ^2^ test at the 0.05 level of statistical significance. 

Another statistical analysis was performed using box and forest plots. Average slopes of growth curve and total integrals for the weight sum of the trunk, giblets, abdominal fat, breast and thigh muscles at 42 days of age (at the slaughter of chickens) in both chicken lines, for both sexes and for all genotypes observed, were evaluated using the laboratory information system Qinslab (Prevention Medicals, Studenka, Czech Republic).

## 3. Results

**Figure 2:**
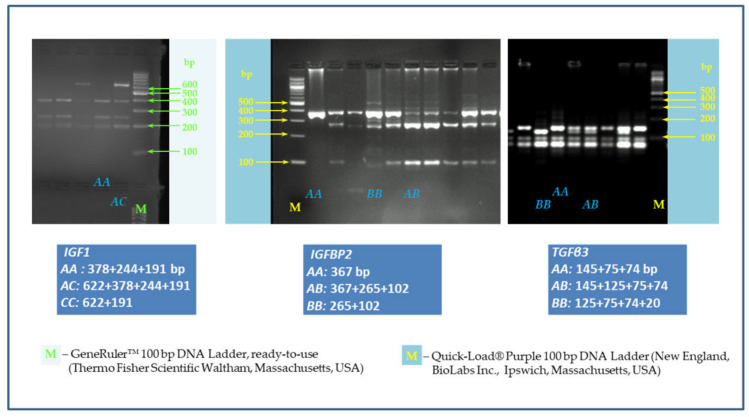
The restriction fragment length polymorphism (RFLP) patterns for *IGF1* (*AA:* 378 + 244 + 191 bp; *AC:* 622 + 378 + 244 + 191 bp; *CC*–it was not detected), *IGFBP2* (*AA*: 367 bp; *AB*: 367 + 265 + 102 bp; *BB:* 265 + 102 bp) and *TGFβ3* (*AA*: 145 + 75 + 74 bp, *AB*: 145 + 125 + 75 + 74 bp; *BB*: 125 + 75 + 74 + 20 bp). Agarose 2%, 120 V, 60 min, Tris-borate-ethylenediaminetetraacetic acid (EDTA) (TBE) buffer. M–marker.

**Figure 3:**
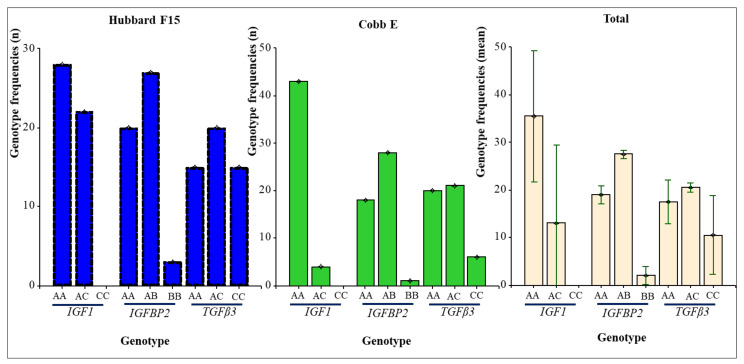
Genotype distribution of individual genes in both chicken lines.

The *IGF1/Hinf*I PCR-RFLP analysis of 97 DNA samples obtained from chicken belonging to broiler lines Hubbard F15 and Cobb E showed only two from three genotypes, namely *AA* (378 + 244 + 191 bp), and *AC* (622 + 378 + 244 + 191 bp), as shown in Figure 2. The *AA* homozygotes (73.20%) predominated over heterozygotes (26.80%)–Table 4, Figure 3. No *CC* homozygous individuals were detected on either of the two broiler lines. Correspondingly, the frequency of allele *A* is much higher (86.60%) than allele *C* (13.40%) in the investigated chicken population, as is evident from Table 4.

In contrast, in *IGFBP2/Eco72*I polymorphism, all three genotypes (*AA, AB*, and *BB*) were found, however, *BB* (265 + 102 bp) homozygotes showed very low frequency (4.12%). The most represented genotype was *AB* (367 + 265 + 102 bp) with a frequency of 56.70%.

Also, in *TGFß3*/*Bsl*I SNP, all three genotypes were detected, with the highest observed genotypic frequency in heterozygotes (42.27%) followed by 36.08% and 21.65% in *AA* (145 + 75 + 74 bp) and *BB (*125 + 75 + 74 + 20 bp), respectively.

Only for IGFBP2 frequencies in total population, Hardy-Weinberg equilibrium (HWE) was identified: *p* < 0.01. 

Table 5, Table 6 and Table 7 show average values of growth performance and carcass traits in both chicken lines, according to individual genotypes. The highest average BW at 42 days was achieved in the Cobb E line, with the *AC* genotype of *IGF1* (2967.50 g), *BB* genotype of *IGFBP2* (3170.00 g) and the *AB* genotype of *TGFβ3* (3104.29 g). The highest average breast muscle (without skin) was found in a Cobb E chicken with an *AC* genotype of *IGF1* (629.25 g), a *BB* genotype of *IGFBP2* (753.00 g) and an *AB* genotype of *TGFβ3* (653.62 g). The Cobb E chicken with an *AC* genotype of *IGF1* (52.50 g) and a *BB* genotype of *IGFBP2* (63.00 g) also had the highest average abdominal fat weight (AFW); whereas for *TGFβ3*, the highest AFW was found in the *AA* genotype (54.95 g). The highest average thigh muscle (with skin) were measured in a Cobb E line chicken with an *AC* genotype of *IGF1* (519.75 g), a *BB* genotype of *IGFBP2* (552.00 g) and an *AB* genotype of *TGFβ3* (538.72 g). On the contrary, the lowest average values of BW at 42 days and AFW showed in the Hubbard F15 line chicken with an *AA* genotype of *IGF1* (2585.00 and 35.32 g, respectively), a *BB* genotype of *IGFBP2* (2413.33 g and 30.33 g, respectively) and an *AA* genotype of *TGFβ3* (2541.33 g and 31.53 g). The lowest breast muscle (without skin) showed the Hubbard F15 line chicken with an *AA* genotype of *IGF1* (501.75 g), an *AA* genotype of *IGFBP2* (498.75 g) and an *AA* genotype of *TGFβ3* (494.33 g). The lowest thigh muscle (with skin) was observed in a Hubbard F15 line chicken with an *AA* genotype of *IGF1 (*470.68 g) and a *BB* genotype of *IGFBP2* (445.00 g) and in a Cobb E line chicken with a *BB* genotype of *TGFβ3* (458.67 g).

By means of statistical software analysis, the relationships between SNPs and individual traits were identified. The fixed effects included in the “whole” model were sex and line, and the *p*-values obtained were adjusted by the number of tests using Bonferroni correction. 

Only the *TGFβ3* SNP (Table 8) resulted in statistical significance for the following parameters: body weight at 21, 28 and 35 days, trunk weight and slaughter value. The *p* values were significant for codominant, dominant and overdominant genetic models, with the exception of the slaughter value, which was not significant for the dominant inheritance pattern.

**Figure 4:**
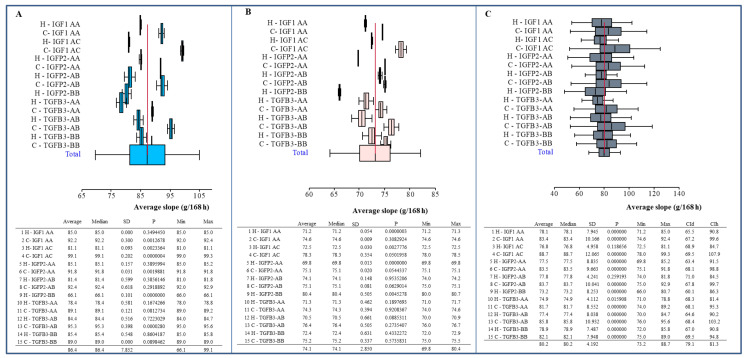
Forest plot of body weight (g) of chicken (at 14–42 days of age) of both lines in individual genotypes. (**A**) Males; (**B**) females; (**C**) total. Statistical characteristics of individual variants: average (n), median, standard deviation (SD), *p*-value (compared to average value), minimum (min) and maximum (max) value. Symbol H is Hubbard F15; C is Cobb E. The line segments represent confidence interval–CI (95%).

Figure 4 shows average slopes of growth curve (14–42 days of age) in individual genotypes of both chicken line and sexes.

**Figure 5:**
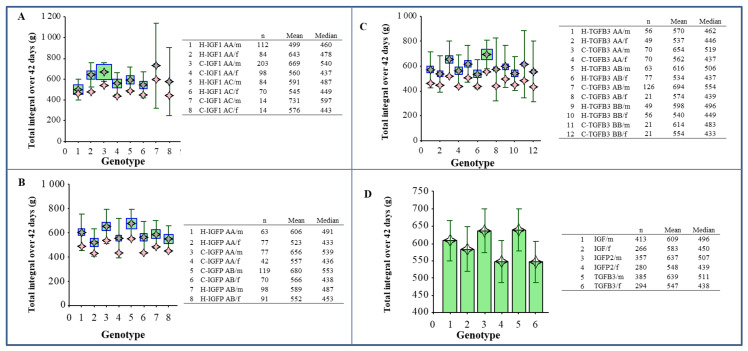
The weight sum of the trunk, giblets, abdominal fat, breast muscle with and without skin and thigh muscle with and without skin. *IGF1* gene with genotype *AA* and *AC* (**A**), *IGFBP2* with genotype *AA* and *AB* (**B**), *TGFβ3* with genotype *AA, AB* and *BB* (**C**). The pink symbol—median, the grey symbol—mean, square indicates the weight (number of samples). A comparison of individual genotypes (**D**). Statistical characteristics of individual variants: number (n), mean and median. The line segments represent confidence interval–CI (95%); m—males, f—females.

Figure 5 represents total integrals for the sum of the trunk, giblets, abdominal fat, breast and thigh muscles at 42 days of age in all genotypes observed.

## 4. Discussion

The study of candidate genes is one of the primary methods to determine whether specific genes are related to economically important traits in farm animals [69]. We performed genotyping of the SNP of three genes linked to consumer-priced characteristics in chicken meat.

One of the main hormones required to normal growth process and muscle development is insulin-like growth factor 1 (IGF-1) [74]. The chicken *IGF1* gene consists of four exons and three introns, spanning more than 50 kb on chromosome 1 [75]. 

*IGF1* encodes the same-name protein (IGF-1), which has a similar molecular structure to insulin [76] and induces insulin-like metabolic effects in muscle and adipose tissues [65]. This protein plays an important role in the proliferation, differentiation and metabolism of myogenic cell lines in chickens [76]. IGF-1 is one of the three ligands (insulin, IGF-1, IGF-2) belonging to the IGF system, which also includes three cell surface binding receptors (InsR, IGF-1R, IGF-2R), and insulin-like growth factor binding proteins (IGF binding proteins, IGFBPs) and IGFBP protease [77]. In addition, the IGF-1 protein is a potent mitogen and an essential stimulus for the differentiation of adipocytes [78]. The production and secretion of IGF-1 is affected by age, nutritional status, and several hormones [79]. The predominant source of IGF-1 is the liver and some other tissues, including muscle, brain and kidney [80]. 

IGF-1 binds to the type 1 insulin-like growth factor receptor (IGF-1R), which plays a critical role in signaling cell survival and proliferation [21]. However, IGF-1 can also bind, albeit with lower affinity, to the insulin receptor [16], regulating some metabolic functions [25].

Insulin-like growth factors (IGFs) provide essential signals for the control of embryonic, as well as postnatal development in vertebrates [81]. In addition to the growth hormone (GH), IGF-1 is one of the two main hormones required to support normal growth in chicken. Optimal growth requires a “set-point” concentration of both IGF-1 and triiodothyronine (T_3_) in blood circulation. Pituitary GH plays a role in controlling the circulating levels of both IGF-1 and T_3_ [74]. IGFs stimulate hepatic glycogen, increase DNA synthesis and promote tissue growth in chicken [82]. The highest level of *IGF1 mRNA* expression was detected in the chicken liver. High levels of *IGF1 mRNA* (10%–30% of the value in the liver) were expressed in spleen, lung and brain of chickens. *IGF1 mRNA* expression was also observed in other extrahepatic tissues such as the kidney, heart, intestine, thymus and muscle of chickens, but these expression levels were less than 4% of that in the liver [83].

The abundant expression of *IGF1* gene was detected in the liver of normal chicken, but no *IGF1 mRNA* expression was found in that organ of dwarf chicken [83]. The expression of hepatic *IGF1 mRNA* level and circulating IGF-1 concentration were significantly higher in chicken with a high growth rate, compared to the line with low growth rate, supporting the hypothesis of its stimulatory effect during post-hatching growth of chickens as stated by Beccavin et al. [84]. The liver is the main site of IGF-1 production during post-hatch growing stages of chicken as described by Kita et al. [85]. 

IGF-1 is significantly altered by the genotype, suggesting a pivotal role in the control of growth rate in broiler chickens [84].

The SNPs within the chicken *IGF1* promoter were reported by numerous previous studies [54,86,87,88,89,90,91,92] but according to the author’s best knowledge, no research work on gene constitution of *IGF1* SNP in the Hubbard F15 and Cobb E lines has been reported until now.

Genotype frequency analysis indicated that the *AA* genotype (73.20%) was of higher frequency than the *AC* (26.80%) and *CC* (0%) genotypes in both chicken lines, which is consistent with another study [92]. For the other two genes (*IGFBP2, TGFβ3*), the predominance of heterozygous (*AB*) genotypes was detected. Interestingly, in Hubbard F15, a distribution of both allele in *TGFβ3* was identical (Table 4).

With regards to the genotype frequencies of *IGF1/Hinf*I gene polymorphism, from the three known restriction patterns, only two genotypes were detected: *AA* and *AC*, with an almost three times higher prevalence of *AA* homozygotes over heterozygotes. The *CC* homozygous genotype was not found in either chicken line, which is consistent with the finding of Moe et al. [90], which reported an absence of the *CC* genotype in two commercial broiler strains (Chunky and Cobb). The noticeable predominance of allele *A* (86.60%) over allele *C* (13.40%) (Table 4) observed in our study is in conspicuous accordance with previous studies. As Moe et al. [90] have shown, allele *C* occurs especially in native chickens, for example in nine Japanese native chicken breeds (Chabo, Ehime-jidori, Gifu-jidori, Koeyoshi, Koshamo, Mikawa, Satsuma-dori, Engie and Tokuchijidori), the frequency of this allele to be 1.0. Our finding of low incidence of *C* allele in broiler chicken also corresponds with the results of genotyping performed by Anh et al. [4], who observed the *CC* genotype of *IGF1* gene with very low frequencies (0.13 to 0.15) in all populations of crossbreds from commercial parent stock broilers with four Thai synthetic chicken lines (the Kaen Thong, Khai Mook Esarn, Soi Nin, and Soi Pet). The *IGF1* SNP gene constitution of these four Thai synthetic chicken lines was studied by Promwatee et al. [92], who found that the *AA* genotype had a considerably lower frequency than the *AC* and *CC* genotypes in all chicken lines except Soi Noi, in which the *AA* and *CC* genotypes were similar. With the exception of Soi Nin, there was the predominance of the *C* allele—the frequency of the *A* allele was lower than that of C in all lines except Soi Noi, where both allelic frequencies were the same. This indicates that allele *C* is evidently typical for native chicken breeds. A higher frequency of allele *A* than that of allele *C* in commercial broiler stocks compared to native chicken can be explained as a result of selection effect on growth traits [90].

It can be concluded that the incidence of a higher *A* allele frequency over *C* allele in the *IGF1* locus observed in our study could be a result of a long-term selection strategy applied in the populations of chosen broiler lines that are the subject of this study.

Various studies reported associations between *IGF1* polymorphism and growth traits in chickens. Zhou et al. [54] and Amills et al. [86] reported that polymorphism of the *IGF1* gene in the promoter and 5′-untranslated region (5′-UTR) was directly associated with chicken growth rate. Bian et al. [89] found that haplotypes based on three *IGF1* polymorphisms (c.-366A > C, c.528G > A and c.*1024C > T–in 5′-flanking, exon 3 and 3′-flanking regions of *IGF1*) were associated with BW traits. 

In our study, *AC* genotype of *IGF1* evinced the highest average BW at 42 days in both chicken lines. This genotype also corresponded with a higher average AFW, breast muscle weight (with or without skin), thigh muscle (with or without skin), slaughter value and slaughter percentage in both lines. On the contrary, the *AA* genotype of *IGF1* was associated with the average lowest BW at 42 days, trunk weight, AFW, breast and thigh muscles, slaughter value and slaughter percentage in both lines. However, no significant difference was identified.

These results are inconsistent with the study of Zhou et al. [54], which observed that broiler line with fragment sizes of 378, 244 and 191 bp (*AA* genotype) showed greater improvement of marketable BW. Additionally, in Thai native chickens, the *AA* genotype resulted in a higher BW compared to the *AC* and *CC* genotypes [93]. Promwatee et al. [92] found, in two synthetic lines (Khai Mook Esarn, Soi Pet), the association between the *AA* genotype and BW at 8 and 12 weeks of age and average daily gain (ADG) at 0–12 and 0–14 weeks. On the contrary, in the Soi Nin synthetic line, BW at 8 and 12 weeks and ADG at 0–12 weeks were associated with the *AC* genotype. In the fourth synthetic line (Kaen Thong), no significant association was found [92]. In Thai native chickens (Chee), the *IGF1* gene was significantly associated with BW at 12 and 16 weeks of age, and ADG during 0–12 and 0–16 weeks of age [94].

IGFBP-2 binds to insulin-like growth factors [64]. IGFBP-2 is the predominant binding protein produced during adipogenesis of white preadipocytes [95]. IGFBP-2 is secreted by white adipocytes and contributes to the prevention of diet-induced obesity [96]. The circulating IGFBP-2 level was significantly and negatively correlated with fasting plasma glucose, triglycerides, low-density lipoprotein (LDL) cholesterol, IGF-1, IGF-2 and insulin C-peptide [97].

IGFBP-2 regulates a broad spectrum of physiological processes involved in growth, development, and differentiation [73]. Both inhibitory and stimulatory effects of IGFBP-2 on cell proliferation have been reported [98]. IGFBP-2 plays an important role in growth and fat metabolism [64]. IGFBP-2 is the predominant IGF binding protein produced during adipogenesis, and is known to increase the insulin-stimulated glucose uptake in myotubes [99]. IGFBP-2 stimulates glucose uptake in a phosphatidylinositol-3-OH kinase (PI3K)-dependent manner. Adipocytes treated with insulin and IGF-1 for 30 min exhibited a significant (*p* < 0.001) increase in PI3K phosphorylation when compared with the control cells. Similarly, IGFBP-2 induced a significant increase in PI3K phosphorylation in 3T3-L1 adipocytes treated for either 30 min (*p* < 0.01) or 24 h (*p* < 0.001). Similarly, IGFBP-2 induced a noticeable increase in AKT phosphorylation in 3T3-L1 adipocytes treated for either 30 min (*p* < 0.05) or 24h (*p* < 0.01) [99]. IGF-1 significantly (*p* < 0.001) increased, whereas insulin failed to induce (*p* > 0.05) AMP-activated protein kinase (AMPK) phosphorylation in 3T3-L1 adipocytes. Similarly, the treatment of adipocytes with IGFBP-2 for either 30 min or 24 h induced a significant (*p* < 0.001) increase in AMPK phosphorylation [99].

Among the seven IGFBPs, IGFBP-2 is the main binding protein secreted by differentiating white preadipocytes, indicating a potential role in the development of obesity. Overexpression of IGFBP-2 was associated with decreased susceptibility to obesity and improved insulin sensitivity [78]. IGFBP-2 expression was associated with fat mass percentage (*p* < 0.02). It was demonstrated that IGFBP-2 is expressed by subcutaneous abdominal adipocytes of obese individuals and that the expression elevated with increasing adiposity and reducing insulin sensitivity [100].

The main functions of IGFBPs are: (1) acting as carrier proteins for circulating IGF-1 and controller of its flow from the vascular space to tissues; (2) increasing IGF-1 half-life and regulating its metabolic clearance [101]; (3) modulating the interaction between IGF-1 and its receptor, and thus indirectly controlling IGF-1 biological activity [102]; (4) modulating IGF-1 in target tissues, inhibiting or activating its specific actions: cell proliferation, differentiation, survival and migration [62,103,104,105]; and (5) providing a specific localization pool of IGF-1, because IGFBPs can associate with cell membranes or the extracellular matrix (ECM) [106]. Moreover, some IGFBPs can possess some biological effects outside the IGF-1 signaling pathways, such as apoptosis induction and proliferation/inhibition in some tumors [105]. 

The *IGFBP2* gene has a total length of 32 kb and it is composed by four exons, 2.0 kb (rat) and 1.6 kb (human) *mRNAs* are generated, and the mature protein is approximately 31 kDa and 36 kDa in rats and humans, respectively [107]. The chicken *IGFBP2* gene spans to more than 38 kb on chromosome 7 (GGA7), consists of four exons, and presents similar organization compared with rats and humans. The chicken *IGFBP2* gene is expressed in a majority of tissues, such as liver, muscle, kidney, heart, ovary, brain, intestine and other tissues [108]. *IGFBP2* gene expression was downregulated in the visceral white adipose tissue of mice, and its circulating levels were reduced in obese mice [96]. Eckstein et al. [109] reported that IGFBP-2 level negatively affected bone size and mineral content in mice, suggesting it was an important regulator of bone biology in vivo.

As for another gene necessary for growth and development processes, the analysis of *IGFBP2/Eco72*I gene polymorphism in the present study showed all three known genotypes (*AA, AB*, and *BB*), with an obvious predominance of the heterozygous genotype (56.70%). However, *BB* (265 + 102 bp) homozygotes showed very low frequency (4.12%). The most represented genotype *AB* (367 + 265 + 102 bp) had a similar frequency to the heterozygotes (53.21%) detected by Li et al. [73], who found almost identical frequencies of both homozygotes (*AA* 22.96%, *BB* 23.83%).

The predominance of *A* allele over *B* allele in *IGFBP2* locus in our study may be, similarly to *IGF1* locus, a long-term selection strategy employed in these chicken populations. 

The study of Li et al. [73] indicated that chicken *IGFBP2* gene intron 2 C1032T (accession number AY 326194) polymorphism was associated with growth and body composition traits in an F_2_ population. Moreover, the *IGFBP2* gene was found to be highly expressed in abdominal fat [73]. QTL for fat deposition was mapped between the marker brackets *LEI0064* and *ROS0019* (75 kb to 27 Mb) on *GGA7* in the chicken linkage map [57], which covers the chicken *IGFBP2* gene (23 to 24 Mb). In Thai native chickens (Chee), the *IGFBP2* gene was significantly associated with body weight at 4 weeks of age, ADG during 0–4 weeks of age and breast width at 16 weeks of age [94].

An excessive abdominal fat in chickens is undesirable and is therefore sought to be reduced, in order to improve the quality of the final product. The IGFBP-2 could inhibit the biological actions of IGF in vivo via endocrine or paracrine mechanisms [22] and indirectly control adipocyte differentiation by regulating the actions of IGF [67]. The structure and function of the *IGFBP2* gene has been analyzed in detail, however, the association of this gene with growth features in chickens has been little studied [66].

In our study, heterozygous genotype *AB* of *IGFBP2* resulted—in both chicken lines—in a higher average BW at 42 days, trunk weight, AFW, breast and thigh muscles, slaughter value and slaughter percentage compared with *AA* genotype (Table 6). On average, chickens with the *IGFBP2-BB* genotype grew slower and simultaneously deposited less fat in the body. These differences, however, were not statistically significant. The lowest breast muscle (without skin) was observed in the Hubbard F15 line chicken with an *AA* genotype of *IGFBP2* (498.75 g). 

The findings of higher BW and AFW in heterozygotes in our research are not consistent with the findings of the study of Li et al. [73], which found that F_2_ chicken homozygous for the *B* allele (*IGFBP2-BB*) had a higher AFW than birds of the other two genotypes. 

The results point to the potential identification of *IGFBP2* as a candidate gene for altering the growth rate and abdominal fat [73]. Reduced growth was associated with increased hepatic *IGFBP2 mRNA* expression and elevated serum IGFBP-2 levels [22], further suggesting IGFBP-2 as a negative growth regulator in vivo [73].

TGF-βs are represented in birds and mammals by three isoforms of secreted cytokines TGF-β1, TGF-β2 and TGF-β3 [70]. The research of Li et al. [69] supported the broad effects of *TGFβ* genes on the growth and development of chickens. Recently, eight from 17 polymorphic sites of the *TGFβ3* gene [53 (T → C), 1653 (C → T), 1755 (A → G), 3343 (C → T), 3540 (C → T), 4786 (C → T), 7263 (C → T) and 7471 (G → A)] have been significantly related to reproduction traits, indicating these polymorphic sites as potential assistant selection markers for improvement of reproductive capacity of Liboyaoshan chicken [110].

The *TGFβ3* gene could be a marker for genetically improving duration fertility in hens. In the recent study performed by Gu et al. [111], four SNPs were identified in intron 1 of *TGFβ3*, and were significantly associated with the duration of fertility in hens (*p* < 0.05). In addition, they identified multi-copy and copy number variants (CNVs) in chicken *TGFβ3*, and later determined significant associations between *TGFβ3* CNVs and duration fertility in hens. Specifically, the *TGFβ3* copy number exhibited a significant positive correlation with its expression (*p* < 0.05). 

A significant association between the *TGFβ3*-*Bsr*I polymorphism and mortality between 14 and 42 days in broiler chickens was reported by Ye et al. [112]. 

In addition, significant effects of *TGFβ3-Bsr*I polymorphism on the cecum content *Salmonella enteritidis* bacterial load were found [113], which could have been of great importance, especially in commercial broiler chicken farms. A moderate association (*p* < 0.17) was found between the *TGFβ3*-*Bsr*I sire allele and antibody response to the *S. enteritidis* vaccine [113]. Polymorphism in the restriction site of *TGFβ3*-*Bsr*I was associated with *S. enteritidis* burden. The heterozygote *A/C* had the highest *S. enteritidis* burden in the cecum, spleen and liver compared with the other two genotypes (*p* < 0.01). The *C/C* genotype of *TGFβ3* showed the lowest bacterial burden for Village Chickens, whereas in Red Junglefowl, the *A/A* genotype exhibited the lowest *S. enteritidis* colonization [114]. *Salmonella enterica* serovar Enteritidis infection is a common concern in poultry production for its negative effects on growth, as well as food safety for humans [114].

In the study of Li et al. [69], the *TGFβ3* polymorphism in broilers crossed with Leghorn was associated with traits of growth and body composition, such as BW, ADG, breast muscle weight, abdominal fat and spleen weight. In our study, for the *TGFβ3* gene, *AB* genotype was the most common in both chicken lines. The allele A was identified as a dominant allele in Cobb E (64.89%), whereas in Hubbard F15, the frequency of both alleles was identical. This finding is different from another study analyzing *TGFβ3* genotypes in breeder hens [115], where the allele *B* was a dominant allele at *TGFβ3* locus, due to it having the highest frequency (0.81).

For the *TGFβ3* gene, different tendencies were observed in the association of the *A* and *B* alleles with the traits observed within both chicken lines. In Cobb E, the *AB* genotype of *TGFβ3* resulted in the highest average BW at 42 days (3104.29 g). The highest AFW had Cobb E chicken with *AA* genotype of *TGFβ3* (54.95 g). On the contrary, in Hubbard F15, the highest average BW at 42 days and AFW were observed in chickens with the *BB* genotype (2618.00 g, 37.33 g, respectively).

The highest average breast muscle (without skin) was found in the *AB* genotype of *TGFβ3* in both lines *(*in Cobb E chicken: 653.62 g and in Hubbard F15: 515.55 g). The highest average thigh muscle (with skin) was also found in the *AB* genotype of *TGFβ3* in both lines, with the highest average value in Cobb E (Cobb E: 538.72 g, Hubbard F15: 476.75 g).

On the contrary, the lowest average value of BW at 42 days was observed in the *AA* genotype of *TGFβ3* in both lines (in the Hubbard F15 line chicken with the *AA* genotype: 2541.33 g and in Cobb E in individuals with the *AA* genotype: 2778.50 g). The lowest AFW was found in the chicken with the *AA* genotype in the Hubbard F15 line (31.53 g), and in Cobb E in birds with the heterozygous genotype *AB* (48.90 g). The lowest breast muscle (without skin) was in the Hubbard F15 line chicken with the *AA* genotype of *TGFβ3* (494.33 g) and in Cobb E in the birds with the *BB* genotype (551.00 g). The lowest thigh muscle (with skin) was observed in the Hubbard F15 line chicken with the *AA* genotype (469.00 g), and in the Cobb E line chicken with the *BB* genotype of *TGFβ3* (458.67 g). 

Association analysis showed that *Bsl*I genotypes of *TGFβ3* are related to some performance traits (Table 8). The statistical analysis revealed a significant association of *TGFβ3* with BW at 21, 28 and 35 days and trunk weight in the codominant (negative value), dominant and overdominant (positive values) genetic model, and with slaughter value in codominant (negative value), recessive and overdominant (positive values) genetic model.

The average slopes of the growth curve (14–42 days of age) constructed according to the line, genotype and sex (Figure 4) was confirmed to be a result of linear growth, as well as lineage and sex differences in body weight. Constructing the graphs of total integrals for the weight sum of the trunk, giblets, abdominal fat, breast and thigh muscles at 42 days of age (at the slaughter of chickens) in both chicken lines (with a separate evaluation of both sexes) and all genotypes observed (Figure 5) showed interesting tendencies. However, no statistically significant dependence was observed, despite apparent differences.

## 5. Conclusions

The presented study demonstrated that the point mutation can affect chicken growth, and confirmed some significant associations between SNP and growth traits. Based on these findings, it can be concluded that the *TGFβ3* gene could be applied as a candidate gene marker for chicken growth traits in the Hubbard F15 and Cobb E broiler line population selection program. However, further association analysis will be required to clarify the effects of this marker on growth and production traits in the broiler chicken population. 

## Figures and Tables

**Table 1:** Light regime in the chicken house.

Day	The Proportion of Light and Darkness Within 24 h	
	Light (h)	Dark (h)
1–7	23	1
8–37	18	6
38	20	4
39	22	2
40–42	23	1

**Table 2:** The content of nutrients in feeding mixtures for broilers (BR1, BR2, BR3) in different periods of the experiment.

Nutrient	Feeding Mixture *		
	BR1	BR2	BR3
	Period		
	1st–10th Day	11th–35th Day	36th–42nd Day
Crude protein (%)	22.01	19.86	18.50
Fat (%)	3.85	5.76	7.64
Lysine (%)	1.16	1.22	1.07
Methionine (%)	0.56	0.56	0.51
Calcium (%)	0.91	0.84	0.81
Phosphorus (%)	0.60	0.54	0.47
Vitamin A (IU/kg)	15,300	12,300	10,300
Vitamin D3 (IU/kg)	5,000	5,000	5,000
Metabolizable energy (MJ/kg)	12.64	13.07	13.59

* The feeding mixtures were produced in ZZN Pelhrimov, a.s., according to given recipes.

**Table 3:** Primers used in polymerase chain reaction (PCR) assay.

Gene		Primer Sequence	Product Length (bp)	Restriction Enzyme	Restriction Enzyme Production Size (bp) *
*IGF1* *SNP/site* *A>C/Promoter and 5′UTR*	forward ^a^	5’-CATTGCGCAGGCTCTATCTG-3´	813	*Hinf*I	*AA: 378+244+191* *AC: 622+378+244+191* *CC: 622+191*
	reverse ^a^	5´-TCAAGAGAAGCCCTTCAAGC-3´			
*IGFBP2* *intron 2 C1032T (accession number AY 326194)*	forward ^b^	5´-GTCCCAGATAAACCTTGCT-3´	367	*Eco72*I	*AA*: 367 *AB:* 367+265+102 *BB:* 265+102
	reverse ^b^	5´-GCTGGCAAGGGGTCTG-3´			
*TGFβ3* *A C/A SNP at base 2,833 (accession number X60091)*	forward ^c^	5´-TCAGGGCAGGTAGAGGGTGT-3´	294	*Bsl*I	*AA:* 145+75+74 *AB:* 145+125+75+74 *BB:* 125+75+74+20
	reverse ^c^	5´-GCCACTGGCAGGATTCTCAC-3´			

^a^ Moody et al. (2003) [72], Zhou et al. (2005) [54]. ^b^ Li et al. (2006) [73]. ^c^ Li et al. (2003) [69]. * All possibilities of fragments.

**Table 4:** Genotype and allele frequencies of *IGF1, IGFBP2* and *TGFβ3* genes in the chicken population.

Broiler Line	Gene	Genotype Frequencies ^a^			Allele Frequencies ^a^		χ^2^ Test HWE ^b^ (*p*-Value)
Hubbard F15	*IGF1*	*AA*	*AC*	*CC*	*A*	*C*	
		28 (56.00)	22 (44.00)	nf	78 (78.00)	22 (22.00)	0.0908
	*IGFBP2*	*AA*	*AB*	*BB*	*A*	*B*	
		20 (40.00)	27 (54.00)	3 (6.00)	67 (67.00)	33 (33.00)	0.1998
	*TGFβ3*	*AA*	*AB*	*BB*	*A*	*B*	
		15 (30.00)	20 (40.00)	15 (30.00)	50 (50.00)	50 (50.00)	0.1639
Cobb E	*IGF1*	*AA*	*AC*	*CC*	*A*	*C*	
		43 (91.49)	4 (8.51)	nf	90 (95.74)	4 (4.26)	1.00
	*IGFBP2*	*AA*	*AB*	*BB*	*A*	*B*	
		18 (38.30)	28 (59.57)	1 (2.13)	64 (68.09)	30 (31.91)	0.0172*
	*TGFβ3*	*AA*	*AB*	*BB*	*A*	*B*	
		20 (42.55)	21 (44.68)	6 (12.77)	61 (64.89)	33 (35.11)	1.00
Total	*IGF1*	*AA*	*AC*	*CC*	*A*	*C*	
		71 (73.20)	26 (26.80)	0 (0)	168 (86.60)	26 (13.40)	0.2066
	*IGFBP2*	*AA*	*AB*	*BB*	*A*	*B*	
		38 (39.18)	55 (56.70)	4 (4.12)	131 (67.53)	63 (32.47)	0.0050*
	*TGFβ3*	*AA*	*AB*	*BB*	*A*	*B*	
		35 (36.08)	41 (42.27)	21 (21.65)	111 (57.22)	83 (42.78)	0.2125

**^a ^**The numbers in brackets are percentage frequencies (relative frequencies). **^b^** HWE–Hardy-Weinberg equilibrium; * statistically significant (*p* < 0.05)*;* nf–not found.

**Table 5:** The average growth performance and carcass traits in the chicken population (according to *IGF1* genotypes).

Parameter	Line					
	Hubbard F15			Cobb E		
	Genotype			Genotype		
	*AA (n = 28)*	*AC (n = 22)*	*CC*	*AA (n = 43)*	*AC (n = 4)*	*CC*
	Mean ± SD; CI					
BW at 14 days *	421.07 ± 32 433; 408	428.18 ± 31 442; 414	-	473.24 ± 40 485; 461	462.50 ± 43 530; 394	-
BW at 21 days *	840.00 ± 69 866; 813	865.00 ± 67 894; 835	-	933.25 ± 96 962; 903	940.00 ± 113 1120; 760	-
BW at 28 days *	1393,21 ± 137 1446; 1340	1436.82 ± 114 1487; 1386	-	1558.84 ± 182 1614; 1502	1552.50 ± 204 1877; 1227	-
BW at 35 days *	1913.21 ± 183 1984; 1842	1947.27 ± 161 2018; 1876	-	2103.95 ± 240 2178; 2030	2152.50 ± 281 2600; 1705	-
BW at 42 days before slaughter *	2585.00 ± 298 2700; 2469	2588.64 ± 265 2706; 2471	-	2919.77 ± 318 3018; 2822	2967.50 ± 415 3627; 2308	-
Trunk weight *	1798.00 ± 190 1871; 1724	1817.45 ± 188 1901; 1734	-	1989.60 ± 224 2058; 1920	2060.25 ± 289 2520; 1600	-
Giblets weight *	156.79 ± 18 163; 149	161.45 ± 14 168; 155	-	169.14 ± 18 174; 163	165.50 ± 21 199; 132	-
Abdominal fat weight *	35.32 ± 9 38; 32	35.68 ± 13 41; 30	-	51.47 ± 12 55; 47	52.50 ± 17 80; 25	-
Breast muscle with skin *	542.18± 53 562; 521	558.00± 64 586; 529	-	668.81± 84 694; 643	684.50± 106 853; 515	-
Breast muscle without skin *	501.75 ± 50 521; 482	517.09 ± 61 544; 489	-	615.98 ± 83 641; 590	629.25 ± 98 712; 530	-
Thigh muscle with skin *	470.68 ± 61 494; 447	475.14 ± 55 499; 450	-	499.98 ± 60 519; 584	519.75 ± 94 669; 371	-
Thigh muscle without skin *	421.39 ± 55 442; 400	426.77 ± 53 450; 402	-	441.88 ± 60 460; 423	464.25 ± 90 608; 320	-
Slaughter value **	69.66	70.22	-	68.14	69.42	-
Slaughter percentage ***	75.74	76.48	-	73.94	75.01	-

BW–average body weight; SD–standard deviation; CI–confidence interval (95%); * (g); ** slaughter value = weight of trunk/BW at 42 days * 100 (%); *** slaughter percentage = weight of trunk+ weight of giblets/BW at 42 days * 100 (%).

**Table 6:** The average growth performance and carcass traits in the chicken population (according to *IGFBP2* genotypes).

Parameter	Line					
	Hubbard F15			Cobb E		
	Genotype			Genotype		
	*AA (20)*	*AB (27)*	*BB (3)*	*AA (17)*	*AB (27)*	*BB (1)*
	Mean ± SD; CI					
BW at 14 days *	419.50 ± 33 435; 403	427.78 ± 32 440; 415	423.33 ± 15 461; 385	468.82 ± 39 489; 449	474.81 ± 43 492; 458	460.00
BW at 21 days *	839.50 ± 72 873; 805	864.07 ± 66 890; 838	810.00 ± 62 965; 655	927.65 ± 95 976; 879	932.22 ± 103 973; 892	1010.00
BW at 28 days *	1392.50 ± 128 1452; 1333	1436.67 ± 128 1487; 1385	1326.67 ± 90 1552; 1101	1535.29 ± 184 1630; 1441	1554.81 ± 184 1628; 1482	1750.00
BW at 35 days *	1894.00 ± 179 1978; 1810	1967.78 ± 164 2032; 1902	1800.00 ± 125 2110; 1489	2096.47 ± 232 2216; 1977	2097.41 ± 256 2199; 1996	2300.00
BW at 42 days before slaughter *	2578.50 ± 320 2728; 2428	2611.85 ± 257 2713; 2510	2413.33± 240 3009; 1818	2891.76 ± 344 3069; 2716	2912.59 ± 311 3036; 2789	3170.00
trunk weight *	1791.20 ± 206 1887; 1695	1827.41 ± 178 1898; 1757	1721.33 ± 157 2110; 1333	1958.06 ± 226 2074; 1842	1996.22 ± 228 2086; 1906	2251.00
giblets weight *	159.45 ± 19 168; 151	159.44 ± 15 165; 154	149.33 ± 18 194; 105	166.53 ± 20 177; 156	168.74 ± 18 176; 162	194.00
abdominal fat weight *	35.55 ± 11 41; 30	36.00 ± 9 40; 32	30.33 ± 18 75; (-14)	50.59 ± 13 57; 44	51.26 ± 12 56; 46	63.00
breast muscle with skin *	539.65 ± 64 569; 510	555.85 ± 56 578; 533	552.00 ± 33 634; 470	651.24 ± 74 689; 613	676.11 ± 90 712; 641	809.00
breast muscle without skin *	498.75 ± 60 527; 471	515.59 ± 53 537; 495	509.67 ± 36 600; 420	598.82 ± 72 636; 562	622.37 ± 89 658; 587	753.00
thigh muscle with skin *	471.80 ± 63 501; 442	476.33 ± 56 498; 454	445.00 ± 52 575; 316	488.06 ± 65 522; 455	503.33 ± 61 527; 479	552.00
thigh muscle without skin *	423.25 ± 59 451; 396	426.74 ± 52 447; 406	400.33 ± 49 522; 278	431.24 ± 63 464; 399	445.56 ± 62 470; 421	487.00
slaughter value **	69.57	69.99	71.36	67.77	68.51	71.01
slaughter percentage ***	75.78	76.12	77.54	73.53	74.31	77.13

BW–average body weight; SD–standard deviation; CI–confidence interval (95%); * (g); ** slaughter value = weight of trunk/BW at 42 days * 100 (%); *** slaughter percentage = weight of trunk + weight of giblets/BW at 42 days * 100 (%).

**Table 7:** The average growth performance and carcass traits in the chicken population (according to *TGFβ3* genotypes).

Parameter	Line					
	Hubbard F15			Cobb E		
	Genotype			Genotype		
	*AA (15)*	*AB (20)*	*BB (15)*	*AA (20)*	*AB (21)*	*BB (6)*
	Mean ± SD; CI					
BW at 14 days *	420.67 ± 40 443; 398	433.00 ± 24 444; 422	416.00 ± 30 433; 399	462.00 ± 38 480; 444	484.76 ± 41 503; 466	463.33 ± 34 499; 428
BW at 21 days *	842.67 ± 72 883; 803	866.00 ± 65 896; 836	839.33 ± 72 879; 799	888.50 ± 93 932; 845	980.00 ± 91 1021; 939	923.33 ± 50 976; 871
BW at 28 days *	1399.33 ± 122 1467; 1332	1420.50 ± 117 1475; 1366	1414.67 ± 154 1499; 1330	1453.00 ± 167 1531; 1375	1664.76 ± 149 1733; 1597	1536.67 ± 125 1668; 1405
BW at 35 days *	1884.67 ± 171 1979; 1790	1938.00 ± 165 2015; 1861	1958.67 ± 187 2062; 1855	1986.00 ± 240 2098; 1874	2241.91 ± 190 2328; 2155	2046.67 ± 176 2231; 1862
BW at 42 days before slaughter *	2541.33 ± 255 2682; 2400	2597.00 ± 302 2738; 2456	2618.00 ± 292 2780; 2456	2778.50 ± 312 2924; 2633	3104.29 ± 270 3227; 2982	2780.00 ± 208 2998; 2562
trunk weight *	1773.27 ± 175 1870; 1676	1825.40 ± 209 1923; 1728	1814.73 ± 177 1913; 1717	1896.95 ± 223 2001; 1793	2129.19 ± 181 2211; 2047	1857.00 ± 136 1999; 1715
giblets weight *	159.00 ± 15 168; 150	158.15 ± 19 167; 149	159.60 ± 15 168; 151	161.45 ± 19 170; 153	177.43 ± 16 185; 170	163.33 ± 14 178; 148
abdominal fat weight *	31.53 ± 7 35; 28	37.05 ± 13 43; 31	37.33 ± 10 43; 32	54.95 ± 14 62; 48	48.90 ± 9 53; 45	49.50 ± 15 66; 33
breast muscle with skin *	533.67 ± 59 566; 501	555.15 ± 68 587; 523	556.60 ± 41 580; 534	648.30 ± 86 689; 608	710.48 ± 72 743; 678	601.83 ± 51 656; 548
breast muscle without skin *	494.33 ± 54 525; 465	515.55 ± 66 546; 485	513.27 ± 39 535; 491	598.60 ± 86 639; 559	653.62 ± 73 687; 621	551.00 ± 52 605; 497
thigh muscle with skin *	469.00 ± 63 504; 434	476.75 ± 63 506; 447	470.80 ± 49 498; 444	475.65 ± 61 504; 447	538.72 ± 51 562; 516	458.67 ± 30 490; 428
thigh muscle without skin *	421.47 ± 56 453; 390	429.10 ± 61 458; 401	418.93 ± 45 444; 394	419.65 ± 61 448; 391	477.05 ± 54 502; 452	407.83 ± 32 441; 375
slaughter value * *	69.80	70.34	69.44	68.26	68.62	66.93
slaughter percentage * * *	76.08	76.43	75.57	74.08	74.34	72.81

BW–average body weight; SD–standard deviation; CI–confidence interval (95%); * (g); ** slaughter value = weight of trunk/BW at 42 days * 100 (%); *** slaughter percentage = weight of trunk + weight of giblets/BW at 42 days * 100 (%).

**Table 8:** Results of statistical analysis for testing association between *TGFβ3* polymorphism and growth performance and carcass traits in the chicken population.

Parameter	Genetic Model				
	Codominant	Dominant	Recessive	Overdominant	Log-Additive
BW at 14 days	ns	ns	ns	ns	ns
BW at 21 days	**−0.013**	**0.014**	0.606	**0.005**	0.199
BW at 28 days	**−0.004**	**0.002**	0.847	**0.004**	**0.035**
BW at 35 days	**−0.004**	**0.001**	0.560	**0.008**	**0.016**
BW at 42 days before slaughter	ns	ns	ns	ns	ns
trunk weight	**−0.010**	**0.013**	0.535	**0.003**	0.215
giblets weight	ns	ns	ns	ns	ns
abdominal fat weight	ns	ns	ns	ns	ns
breast muscle with skin	ns	ns	ns	ns	ns
breast muscle without skin	ns	ns	ns	ns	ns
thigh muscle with skin	ns	ns	ns	ns	ns
thigh muscle without skin	ns	ns	ns	ns	ns
slaughter value	**−0.015**	0.646	**0.015**	**0.014**	0.306
slaughter percentage	ns	ns	ns	ns	ns

BW–body weight; ns–no significant SNP after Bonferroni correction. Statistical significances at significance level 0.05 are highlighted in bold.

## Abbreviations

ADG	average daily gain
AFW	abdominal fat weight
AKT	serine/threonine kinase
AMP	adenosine monophosphate
AMPK	AMP-activated protein kinase
BACs	bacterial artificial chromosomes
BAD	BCL-2 antagonist of cell death
BCL-2	antiapoptotic B-cell lymphoma/leukemia-2
bp	base pairs
BW	body weight
CI	confidence interval
CNVs	copy number variants
cM	centimorgan
DNA	deoxyribonucleic acid
ECM	extracellular matrix
EDTA	ethylenediaminetetraacetic acid
ERK	extracellular signal-regulated kinase
FOXO	Forkhead box, class O; subfamily of Forkhead transcription factors
Gb	giga base pairs
GGA1, GGA7	Gallus gallus autosome 1, Gallus gallus autosome 7
GH	growth hormone
GSK-3β	glycogen synthase kinase-3β
HWE	Hardy-Weinberg equilibrium
IGF	insulin-like growth factor
IGF-1	insulin-like growth factor 1
IGF1	insulin-like growth factor 1 gene
IGF-1R	the type 1 insulin-like growth factor receptor
IGF-2R	the type 2 insulin-like growth factor receptor
IGFBP	insulin-like growth factor binding protein
IGFBP-2, IGFBP-3	insulin-like growth factor binding protein 2, 3
IGFBP2	insulin-like growth factor binding protein 2 gene
InsR	insulin receptor
IRS	insulin receptor substrate
IU	international units
kb	kilobase pairs
LDL	low-density lipoprotein
MAP	mitogen-activated protein
MAPK	mitogen-activated protein kinase
Mb	megabase pairs
MEK	mitogen-activated protein kinase kinase 7 (MAP2K7)
MMAS	molecular marker-assisted selection
mRNA	messenger RNA
mTOR	mammalian target of rapamycin
mTOR-C1, mTOR-C2	complexes of mTOR
p27	a protein of 27 kDa that regulates the cell cycle
PI3K	phosphatidylinositol-3-OH kinase
PCR	polymerase chain reaction
PDK1	3-phosphoinositide-dependent protein kinase-1
QTL	quantitative trait locus
RAF	proto-oncogene serine/threonine-protein kinase
RAS	a protein playing a key role in signal transduction of cell growth and differentiation
RFLP	restriction fragment length polymorphism
S6K-1/2	ribosomal protein S6 kinase 1/2
SD	standard deviation
SHC	the SRC homology and collagen protein
SMADs	proteins that are the main signal transducers for receptors of TGFß
SNP	single nucleotide polymorphism
SRC	proto-oncogene tyrosine-protein kinase
T_3_	triiodothyronine
TBE buffer	Tris-borate-EDTA buffer
TGFß	transforming growth factor β
TGFß3	transforming growth factor β3 gene
TSC-1/2	tuberous sclerosis protein ½
4E-BP1	eukaryotic translation initiation factor 4E (eIF4E)-binding protein 1

## References

[1] Godfray H.C.J., Aveyard P., Garnett T., Hall J.W., Key T.J., Lorimer J., Pierrehumbert R.T., Scarborough P., Springmann M., Jebb S.A. (2018). Meat consumption, health, and the environment. Science.

[2] FAO FAOSTAT: Food Supply - Livestock and Fish Primary Equivalent. http://www.fao.org/faostat/en/#data/CL/visualize.

[3] Kim H.-E., Lee J.-J., Lee M.-J., Kim B.-S. (2019). Analysis of microbiome in raw chicken meat from butcher shops and packaged products in South Korea to detect the potential risk of foodborne illness. Food Res. Int..

[4] Anh N.T.L., Kunhareang S., Duangjinda M. (2015). Association of chicken growth hormones and insulin-like growth factor gene polymorphisms with growth performance and carcass traits in Thai broilers. Asian-Australas. J. Anim. Sci..

[5] Zhang C., Zhang W., Luo H., Yue W., Gao M., Jia Z. (2008). A new single nucleotide polymorphism in the IGF-I gene and its association with growth traits in the Nanjiang Huang goat. Asian-Australas. J. Anim. Sci..

[6] Hillier L., Miller W., Birney E., Warren W., Hardison R., Ponting C., Bork P., Burt D., Groenen M., Delany M. (2004). International Chicken Genome Sequencing Consortium: Sequence and comparative analysis of the chicken genome provide unique perspectives on vertebrate evolution. Nature.

[7] Bellott D.W., Skaletsky H., Pyntikova T., Mardis E.R., Graves T., Kremitzki C., Brown L.G., Rozen S., Warren W.C., Wilson R.K. (2010). Convergent evolution of chicken Z and human X chromosomes by expansion and gene acquisition. Nature.

[8] Rubin C.-J., Zody M.C., Eriksson J., Meadows J.R., Sherwood E., Webster M.T., Jiang L., Ingman M., Sharpe T., Ka S. (2010). Whole-genome resequencing reveals loci under selection during chicken domestication. Nature.

[9] Schmid M., Smith J., Burt D.W., Aken B.L., Antin P.B., Archibald A.L., Ashwell C., Blackshear P.J., Boschiero C., Brown C.T. (2015). Third report on chicken genes and chromosomes 2015. Cytogenet. Genome Res..

[10] Warren W.C., Hillier L.W., Tomlinson C., Minx P., Kremitzki M., Graves T., Markovic C., Bouk N., Pruitt K.D., Thibaud-Nissen F. (2017). A new chicken genome assembly provides insight into avian genome structure. G3 Bethesda.

[11] Reyer H., Hawken R., Murani E., Ponsuksili S., Wimmers K. (2015). The genetics of feed conversion efficiency traits in a commercial broiler line. Sci. Rep..

[12] Dekkers J.C. (2004). Commercial application of marker-and gene-assisted selection in livestock: strategies and lessons. J. Anim. Sci..

[13] Zhang X., Jiang X., Liu Y., Du H., Zhu Q. (2007). Identification of Ava I polymorphisms in the third intron of GH gene and their associations with abdominal fat in chickens. Poult. Sci..

[14] Fontanesi L., Scotti E., Tazzoli M., Beretti F., Dall’Olio S., Davoli R., Russo V. (2007). Investigation of allele frequencies of the growth hormone receptor (GHR) F279Y mutation in dairy and dual purpose cattle breeds. Ital. J. Anim. Sci..

[15] Jung H.J., Suh Y. (2015). Regulation of IGF-1 signaling by microRNAs. Front. Genet..

[16] Laron Z. (2001). Insulin-like growth factor 1 (IGF-1): a growth hormone. Mol. Pathol..

[17] LeRoith D. (1991). Insulin-like growth factors: molecular and cellular aspects.

[18] Ohlsson C., Sjögren K., Jansson J.-O., Isaksson O. (2000). The relative importance of endocrine versus autocrine/paracrine insulin-like growth factor-I in the regulation of body growth. Pediatr. Nephrol..

[19] Wang Y., Bikle D.D., Chang W. (2013). Autocrine and paracrine actions of IGF-I signaling in skeletal development. Bone Res..

[20] Chrysis D., Calikoglu A.S., Ye P., D’Ercole A.J. (2001). Insulin-like growth factor-I overexpression attenuates cerebellar apoptosis by altering the expression of Bcl family proteins in a developmentally specific manner. J. Neurosci..

[21] Galvan V., Logvinova A., Sperandio S., Ichijo H., Bredesen D.E. (2003). Type 1 insulin-like growth factor receptor (IGF-IR) signaling inhibits apoptosis signal-regulating kinase 1 (ASK1). J. Biol. Chem..

[22] Hoeflich A., Wu M., Mohan S., Föll J., Wanke R.D., Froehlich T., Arnold G.J., Lahm H., Kolb H.J., Wolf E. (1999). Overexpression of insulin-like growth factor-binding protein-2 in transgenic mice reduces postnatal body weight gain. Endocrinology.

[23] Rajaram S., Baylink D.J., Mohan S. (1997). Insulin-like growth factor-binding proteins in serum and other biological fluids: regulation and functions. Endocr. Rev..

[24] NIH National Institutes of Health. Genetics Home Reference..

[25] Rocio G., Morales-Garza L.A., Martin-Estal I., Castilla-Cortazar I. (2017). Insulin-Like Growth Factor-1 Deficiency and Cirrhosis Establishment. J. Clin. Med. Res..

[26] Denduluri S.K., Idowu O., Wang Z., Liao Z., Yan Z., Mohammed M.K., Ye J., Wei Q., Wang J., Zhao L. (2015). Insulin-like growth factor (IGF) signaling in tumorigenesis and the development of cancer drug resistance. Genes Dis..

[27] Clemmons D.R. (2018). 40 YEARS OF IGF1: Role of IGF-binding proteins in regulating IGF responses to changes in metabolism. J. Mol. Endocrinol..

[28] Aguirre G., De Ita J.R., De La Garza R., Castilla-Cortazar I. (2016). Insulin-like growth factor-1 deficiency and metabolic syndrome. J. Transl. Med..

[29] De Ita J.R., Castilla-Cortázar I., Aguirre G., Sanchez-Yago C., Santos-Ruiz M.O., Guerra-Menendez L., Martin-Estal I., García-Magariño M., Lara-Díaz V.J., Puche J. (2015). Altered liver expression of genes involved in lipid and glucose metabolism in mice with partial IGF-1 deficiency: an experimental approach to metabolic syndrome. J. Transl. Med..

[30] Desbois-Mouthon C., Wendum D., Cadoret A., Rey C., Leneuve P., Blaise A., Housset C., Tronche F., Le Bouc Y., Holzenberger M. (2006). Hepatocyte proliferation during liver regeneration is impaired in mice with liver-specific IGF-1R knockout. FASEB J..

[31] García-Fernández M., Castilla-Cortázar I., Díaz-Sánchez M., Díez Caballero F., Castilla A., Diaz Casares A., Varela-Nieto I., González-Barón S. (2003). Effect of IGF-I on total serum antioxidant status in cirrhotic rats. J. Physiol. Biochem..

[32] García-Fernández M., Castilla-Cortázar I., Díaz-Sanchez M., Navarro I., Puche J.E., Castilla A., Casares A.D., Clavijo E., González-Barón S. (2005). Antioxidant effects of insulin-like growth factor-I (IGF-I) in rats with advanced liver cirrhosis. BMC Gastroenterol..

[33] Fernández A.M., Kim J.K., Yakar S., Dupont J., Hernandez-Sanchez C., Castle A.L., Filmore J., Shulman G.I., Le Roith D. (2001). Functional inactivation of the IGF-I and insulin receptors in skeletal muscle causes type 2 diabetes. Genes Dev..

[34] Héron-Milhavet L., Haluzik M., Yakar S., Gavrilova O., Pack S., Jou W.C., Ibrahimi A., Kim H., Hunt D., Yau D. (2004). Muscle-specific overexpression of CD36 reverses the insulin resistance and diabetes of MKR mice. Endocrinology.

[35] Higashi Y., Sukhanov S., Anwar A., Shai S.-Y., Delafontaine P. (2012). Aging, atherosclerosis, and IGF-1. J. Gerontol. Ser. A Biomed. Sci. Med. Sci..

[36] Jacob R., Barrett E., Plewe G., Fagin K.D., Sherwin R.S. (1989). Acute effects of insulin-like growth factor I on glucose and amino acid metabolism in the awake fasted rat. Comparison with insulin. J. Clin. Invest..

[37] Jallali N., Ridha H., Thrasivoulou C., Butler P., Cowen T. (2007). Modulation of intracellular reactive oxygen species level in chondrocytes by IGF-1, FGF, and TGF-β1. Connect. Tissue Res..

[38] Kelley K.W., Weigent D.A., Kooijman R. (2007). Protein hormones and immunity. Brain Behav. Immun..

[39] Kudo Y., Iwashita M., Iguchi T., Takeda Y. (1996). The regulation of L-proline transport by insulin-like growth factor-I in human osteoblast-like SaOS-2 cells. Pflugers Arch..

[40] Laager R., Ninnis R., Keller U. (1993). Comparison of the effects of recombinant human insulin-like growth factor-I and insulin on glucose and leucine kinetics in humans. J. Clin. Invest..

[41] Locatelli V., Bianchi V.E. (2014). Effect of GH/IGF-1 on bone metabolism and osteoporosis. Int. J. Endocrinol..

[42] Mauras N., O’Brien K.O., Welch S., Rini A., Helgeson K., Vieira N.E., Yergey A.L. (2000). Insulin-like growth factor I and growth hormone (GH) treatment in GH-deficient humans: differential effects on protein, glucose, lipid, and calcium metabolism. J. Clin. Endocrinol. Metab..

[43] Moxley 3rd R., Arner P., Moss A., Skottner A., Fox M., James D., Livingston J.N. (1990). Acute effects of insulin-like growth factor I and insulin on glucose metabolism in vivo. Am. J. Physiol.-Endoc. M..

[44] Muguerza B., Castilla-Cortázar I., García M.a., Quiroga J., Santidrián S., Prieto J. (2001). Antifibrogenic effect in vivo of low doses of insulin-like growth factor-I in cirrhotic rats. Biochim. Biophys. Acta Mol. Basis Dis..

[45] Pennisi P.A., Kopchick J.J., Thorgeirsson S., LeRoith D., Yakar S. (2004). Role of growth hormone (GH) in liver regeneration. Endocrinology.

[46] Pennisi P., Gavrilova O., Setser-Portas J., Jou W., Santopietro S., Clemmons D., Yakar S., LeRoith D. (2006). Recombinant human insulin-like growth factor-I treatment inhibits gluconeogenesis in a transgenic mouse model of type 2 diabetes mellitus. Endocrinology.

[47] Powell-Braxton L., Hollingshead P., Warburton C., Dowd M., Pitts-Meek S., Dalton D., Gillett N., Stewart T.A. (1993). IGF-I is required for normal embryonic growth in mice. Genes Dev..

[48] Pratipanawatr T., Pratipanawatr W., Rosen C., Berria R., Bajaj M., Cusi K., Mandarino L., Kashyap S., Belfort R., DeFronzo R.A. (2002). Effect of IGF-I on FFA and glucose metabolism in control and type 2 diabetic subjects. Am. J. Physiol. Endoc. M..

[49] Puche J.E., García-Fernández M.a., Muntané J., Rioja J., Gonzalez-Baron S., Castilla Cortazar I. (2008). Low doses of insulin-like growth factor-I induce mitochondrial protection in aging rats. Endocrinology.

[50] Thorén M.C., Wivall-Helleryd I.-L., Blum W.F., Hall K.E. (1994). Effects of repeated subcutaneous administration of recombinant human insulin-like growth factor I in adults with growth hormone deficiency. Eur. J. Endocrinol..

[51] Tu W., Cheung P.-T., Lau Y.-L. (2000). Insulin-like growth factor 1 promotes cord blood T cell maturation and inhibits its spontaneous and phytohemagglutinin-induced apoptosis through different mechanisms. J. Immunol..

[52] Vincent A.M., Feldman E.L. (2002). Control of cell survival by IGF signaling pathways. Growth Horm. IGF Res..

[53] Walsh P.T., Smith L.M., O’Connor R. (2002). Insulin-like growth factor-1 activates Akt and Jun N-terminal kinases (JNKs) in promoting the survival of T lymphocytes. Immunology.

[54] Zhou H., Mitchell A., McMurtry J., Ashwell C., Lamont S.J. (2005). Insulin-like growth factor-I gene polymorphism associations with growth, body composition, skeleton integrity, and metabolic traits in chickens. Poult. Sci..

[55] Bikle D.D., Tahimic C., Chang W., Wang Y., Philippou A., Barton E.R. (2015). Role of IGF-I signaling in muscle bone interactions. Bone.

[56] Sewalem A., Morrice D., Law A., Windsor D., Haley C., Ikeobi C., Burt D., Hocking P. (2002). Mapping of quantitative trait loci for body weight at three, six, and nine weeks of age in a broiler layer cross. Poult. Sci..

[57] Ikeobi C., Woolliams J., Morrice D., Law A., Windsor D., Burt D., Hocking P. (2002). Quantitative trait loci affecting fatness in the chicken. Anim. Genet..

[58] Bach L.A., Headey S.J., Norton R.S. (2005). IGF-binding proteins–the pieces are falling into place. Trends Endocrinol. Metab..

[59] Clemmons D.R. (2016). Role of IGF binding proteins in regulating metabolism. Trends Endocrinol. Metab..

[60] Kutsukake M., Ishihara R., Momose K., Isaka K., Itokazu O., Higuma C., Matsutani T., Matsuda A., Sasajima K., Hara T. (2008). Circulating IGF-binding protein 7 (IGFBP7) levels are elevated in patients with endometriosis or undergoing diabetic hemodialysis. Reprod. Biol. Endocrin..

[61] Russo V., Gluckman P., Feldman E., Werther G. (2005). The insulin-like growth factor system and its pleiotropic functions in brain. Endocr. Rev..

[62] Jones J.I., Clemmons D.R. (1995). Insulin-like growth factors and their binding proteins: biological actions. Endocr. Rev..

[63] Kita K., Nagao K., Taneda N., Inagaki Y., Hirano K., Shibata T., Yaman M.A., Conlon M.A., Okumura J.-i. (2002). Insulin-like growth factor binding protein-2 gene expression can be regulated by diet manipulation in several tissues of young chickens. J. Nutr..

[64] Leng L., Wang S., Li Z., Wang Q., Li H. (2009). A polymorphism in the 3′-flanking region of insulin-like growth factor binding protein 2 gene associated with abdominal fat in chickens. Poult. Sci..

[65] Monzavi R., Cohen P. (2002). IGFs and IGFBPs: role in health and disease. Best Pract. Res. Clin. Endocrinol. Metab..

[66] Zhao X., Li M., Xu S., Liu G. (2015). Single Nucleotide Polymorphisms in IGFBP-2 Gene and Their Associations with Body Weight Traits on Jinghai Yellow Chicken. Rev. Bras. Cienc. Avic..

[67] Richardson R., Hausman G., Wright J. (1998). Growth factor regulation of insulin-like growth factor (IGF) binding proteins (IGFBP) and preadipocyte differentiation in porcine stromal-vascular cell cultures. Growth Dev. Aging..

[68] Butterwith S., Goddard C. (1991). Regulation of DNA synthesis in chicken adipocyte precursor cells by insulin-like growth factors, platelet-derived growth factor and transforming growth factor-β. J. Endocrinol..

[69] Li H., Deeb N., Zhou H., Mitchell A., Ashwell C., Lamont S.J. (2003). Chicken quantitative trait loci for growth and body composition associated with transforming growth factor-beta genes. Poult. Sci..

[70] Wu M.Y., Hill C.S. (2009). TGF-β superfamily signaling in embryonic development and homeostasis. Dev. Cell.

[71] Xu X., Zheng L., Yuan Q., Zhen G., Crane J.L., Zhou X., Cao X. (2018). Transforming growth factor-β in stem cells and tissue homeostasis. Bone Res..

[72] Moody D., Haynie J., Schreiweis M., Hester P. Identification of SNP in candidate genes for osteoporosis in chickens. Proceedings of the Plant and Animal Genome XI.

[73] Li Z., Li H., Zhang H., Wang S., Wang Q., Wang Y. (2006). Identification of a single nucleotide polymorphism of the insulin-like growth factor binding protein 2 gene and its association with growth and body composition traits in the chicken. J. Anim. Sci..

[74] Scanes C.G. (2009). Perspectives on the endocrinology of poultry growth and metabolism. Gen. Comp. Endocrinol..

[75] Kajimoto Y., Rotwein P. (1991). Structure of the chicken insulin-like growth factor I gene reveals conserved promoter elements. J. Biol. Chem..

[76] Duclos M. (2005). Insulin-like growth factor-I (IGF-1) mRNA levels and chicken muscle growth. J. Physiol. Pharmacol..

[77] Li P., Sun X., Cai G., Chen X. (2017). Insulin-like growth factor system and aging. J. Aging. Sci..

[78] Wheatcroft S.B., Kearney M.T., Shah A.M., Ezzat V.A., Miell J.R., Modo M., Williams S.C., Cawthorn W.P., Medina-Gomez G., Vidal-Puig A. (2007). IGF-binding protein-2 protects against the development of obesity and insulin resistance. Diabetes.

[79] Skottner A. (2012). Biosynthesis of growth hormone and insulin-like growth factor-I and the regulation of their secretion. Open Endocrinol. J..

[80] Barton E.R. (2006). The ABCs of IGF-I isoforms: impact on muscle hypertrophy and implications for repair. Appl. Physiol. Nutr. Me..

[81] Dupont J., Holzenberger M. (2003). Biology of insulin-like growth factors in development. Birth Defects Res. C Embryo Today.

[82] McMurtry J., Francis G., Upton Z. (1997). Insulin-like growth factors in poultry. Domest. Anim. Endocrinol..

[83] Tanaka M., Hayashida Y., Sakaguchi K., Ohkubo T., Wakita M., Hoshino S., Nakashima K. (1996). Growth hormone-independent expression of insulin-like growth factor I messenger ribonucleic acid in extrahepatic tissues of the chicken. Endocrinology.

[84] Beccavin C., Chevalier B., Cogburn L., Simon J., Duclos M. (2001). Insulin-like growth factors and body growth in chickens divergently selected for high or low growth rate. J. Endocrinol..

[85] Kita K., Nagao K., Okumura J. (2005). Nutritional and tissue specificity of IGF-I and IGFBP-2 gene expression in growing chickens-A review. Asian-Australas. J. Anim. Sci..

[86] Amills M., Jimenez N., Villalba D., Tor M., Molina E., Cubilo D., Marcos C., Francesch A., Sanchez A., Estany J. (2003). Identification of three single nucleotide polymorphisms in the chicken insulin-like growth factor 1 and 2 genes and their associations with growth and feeding traits. Poult. Sci..

[87] Bennett A., Hester P., Spurlock D. (2006). Polymorphisms in vitamin D receptor, osteopontin, insulin-like growth factor 1 and insulin, and their associations with bone, egg and growth traits in a layer–broiler cross in chickens. Anim. Genet..

[88] Bhattacharya T., Chatterjee R., Dushyanth K., Paswan C., Shukla R., Shanmugam M. (2015). Polymorphism and expression of insulin-like growth factor 1 (IGF1) gene and its association with growth traits in chicken. Br. Poult. Sci..

[89] Bian L., Wang S., Wang Q., Zhang S., Wang Y., Li H. (2008). Variation at the insulin-like growth factor 1 gene and its association with body weight traits in the chicken. J. Anim. Breed. Genet..

[90] Moe H.H., Shimogiri T., Kawabe K., Nishibori M., Okamoto S., Hashiguchi T., Maeda Y. (2009). Genotypic frequency in Asian native chicken populations and gene expression using insulin-like growth factor 1 (IGF1) gene promoter polymorphism. J. Poult. Sci..

[91] Nagaraja S., Aggrey S., Yao J., Zadworny D., Fairfull R., Kuhnlein U. (2000). Brief communication. Trait association of a genetic marker near the IFG-I gene in egg-laying chickens. J. Hered..

[92] Promwatee N., Laopaiboon B., Vongpralub T., Phasuk Y., Kunhareang S., Boonkum W., Duangjinda M. (2013). Insulin-like growth factor I gene polymorphism associated with growth and carcass traits in Thai synthetic chickens. Genet. Mol. Res..

[93] Promwatee N., Duangjinda M. Association of Single Nucleotide Polymorphisms in GHSR, IGF-I, cGH and IGFBP2 Gene with Growth Traits in Thai Native Chickens. Proceedings of the 14th AAAP Animal Science Congress.

[94] Promwatee N., Duangjinda M., Boonkum W., Loapaiboon B. (2011). Association of single nucleotide polymorphisms in GHSR, IGFI, cGH and IGFBP2 genes on growth traits in Thai Native Chickens (Chee and Pradu Hang Dam). Khon Kaen Agr. J..

[95] Yau S.W., Russo V.C., Clarke I.J., Dunshea F.R., Werther G.A., Sabin M.A. (2015). IGFBP-2 inhibits adipogenesis and lipogenesis in human visceral, but not subcutaneous, adipocytes. Int. J. Obes..

[96] Li Z., Picard F. (2010). Modulation of IGFBP2 mRNA expression in white adipose tissue upon aging and obesity. Horm. Metab. Res..

[97] Heald A., Kaushal K., Siddals K., Rudenski A., Anderson S., Gibson J. (2006). Insulin-like growth factor binding protein-2 (IGFBP-2) is a marker for the metabolic syndrome. Exp. Clin. Endocrinol. Diabetes.

[98] Hoeflich A., Reisinger R., Lahm H., Kiess W., Blum W.F., Kolb H.J., Weber M.M., Wolf E. (2001). Insulin-like growth factor-binding protein 2 in tumorigenesis: protector or promoter?. Cancer Res..

[99] Assefa B., Mahmoud A.M., Pfeiffer A.F., Birkenfeld A.L., Spranger J., Arafat A.M. (2017). Insulin-like growth factor (IGF) binding protein-2, independently of IGF-1, induces GLUT-4 translocation and glucose uptake in 3T3-L1 adipocytes. Oxid. Med. Cell Longev..

[100] Claudio M., Benjamim F., Riccardo B., Massimiliano C., Francesco B., Luciano C. (2010). Adipocytes IGFBP-2 expression in prepubertal obese children. Obesity.

[101] Hwa V., Oh Y., Rosenfeld R.G. (1999). The insulin-like growth factor-binding protein (IGFBP) superfamily. Endocr. Rev..

[102] Conchillo M., Prieto J., Quiroga J. (2007). Insulin-like growth factor I (IGF-I) and liver cirrhosis. Rev. Esp. Enferm. Dig. Madrid.

[103] Firth S.M., Baxter R.C. (2002). Cellular actions of the insulin-like growth factor binding proteins. Endocr. Rev..

[104] Yin P., Xu Q., Duan C. (2004). Paradoxical actions of endogenous and exogenous insulin-like growth factor-binding protein-5 revealed by RNA interference analysis. J. Biol. Chem..

[105] Duan C., Xu Q. (2005). Roles of insulin-like growth factor (IGF) binding proteins in regulating IGF actions. Gen. Comp. Endocrinol..

[106] Oh Y., Nagalla S.R., Yamanaka Y., Kim H.-S., Wilson E., Rosenfeld R.G. (1996). Synthesis and characterization of insulin-like growth factor-binding protein (IGFBP)-7 Recombinant human mac25 protein specifically binds IGF-I and-II. J. Biol. Chem..

[107] Shimasaki S., Ling N. (1991). Identification and molecular characterization of insulin-like growth factor binding proteins (IGFBP-1,-2,-3,-4,-5 and-6). Prog. Growth Factor Res..

[108] Schoen T., Mazuruk K., Waldbillig R., Potts J., Beebe D., Chader G., Rodriguez I. (1995). Cloning and characterization of a chick embryo cDNA and gene for IGF-binding protein-2. J. Mol. Endocrinol..

[109] Eckstein F., Pavicic T., Nedbal S., Schmidt C., Wehr U., Rambeck W., Wolf E., Hoeflich A. (2002). Insulin-like growth factor-binding protein-2 (IGFBP-2) overexpression negatively regulates bone size and mass, but not density, in the absence and presence of growth hormone/IGF-I excess in transgenic mice. Anat. Embryol..

[110] Wu S., Zhu L., Tang J., Wu G. (2017). TGFβ3 gene polymorphism and correlations between TGFβ3 gene and reproduction traits in Liboyaoshan chicken. Southwest. China J. Agric. Sci..

[111] Gu L., Sun C., Gong Y., Yu M., Li S. (2017). Novel copy number variation of the TGFβ3 gene is associated with TGFβ3 gene expression and duration of fertility traits in hens. PLoS ONE.

[112] Ye X., Avendano S., Dekkers J., Lamont S. (2006). Association of twelve immune-related genes with performance of three broiler lines in two different hygiene environments. Poult. Sci..

[113] Malek M., Lamont S.J. (2003). Association of INOS, TRAIL, TGF-β2, TGF-β3, and IgL genes with response to Salmonella enteritidis in poultry. Genet Sel. Evol..

[114] Tohidi R., Idris I., Malar Panandam J., Hair Bejo M. (2013). The effects of polymorphisms in 7 candidate genes on resistance to Salmonella Enteritidis in native chickens. Poult. Sci..

[115] Enayati B., Rahimi-Mianji G. (2009). Genomic growth hormone, growth hormone receptor and transforming growth factor β-3 gene polymorphism in breeder hens of Mazandaran native fowls. Afr. J. Biotechnol..

